# Phenotypic Identification, Genetic Characterization, and Selective Signal Detection of Huitang Duck

**DOI:** 10.3390/ani14121747

**Published:** 2024-06-10

**Authors:** Haojie Ma, Bingjin Lin, Zhiyao Yan, Yueyue Tong, Huichao Liu, Xi He, Haihan Zhang

**Affiliations:** 1 College of Animal Science and Technology, Hunan Agricultural University, Changsha 410128, China; haojie210@126.com (H.M.); 13435111244@163.com (B.L.); yanzhiyao983@163.com (Z.Y.); ty9824@126.com (Y.T.); hcliu1@126.com (H.L.); hexi@hunau.edu.cn (X.H.); 2 Hunan Engineering Research Center of Poultry Production Safety, Changsha 410128, China; 3 Ministry of Education Engineering Research Center of Feed Safety and Efficient Use, Changsha 410128, China

**Keywords:** Huitang duck, phenotype, population structure, selective sweep

## Abstract

**Simple Summary:**

The aim of this study was to explore the genetic resources of the Huitang duck (HT) in Hunan Province, China, and investigate its population structure and genetic potential through comparative analysis with various duck populations. The results showed that HT belonged to the versatile duck breed, exhibiting significant phenotypic and genetic differences compared to other duck breeds in Hunan Province. The inspection of genomic selective signals in their genome showed that selected candidate genes were involved in the growth and development of skeletal muscle and ovary. These findings provide valuable information for future breeding programs and genetic preservation of HT.

**Abstract:**

The Huitang duck (HT), a long-domesticated elite local breed from Hunan Province, China, with excellent meat quality, has not had its population genetic structure and genomic selective sweeps extensively studied to date. This study measured the phenotypic characteristics of HT and conducted comparative analysis between HT and 16 different duck breeds, including wild, indigenous, and meat breeds, to characterize its population structure and genetic potential. The results revealed that HT is a dual-purpose indigenous breed with a genetic background closely related to the Youxian sheldrake and Linwu ducks. In the selective sweep analysis between HT and Linwu ducks, genes such as *PLCG2*, *FN1,* and *IGF2BP2*, which are associated with muscle growth and development, were identified near the 27 selection signals. The comparison between HT and Jinding ducks revealed 68 selective signals that contained important genes associated with ovarian development (*GRIK4*, *MAP3K8*, and *TGIF1*) and egg-laying behaviors (*ERBB4*). Selective sweep analysis between HT and Youxian sheldrake ducks found 93 selective regions covering genes related to both meat (*IGF1R* and *IGFBP5*) and egg-production (*FOXO3* and *ITPR1*) traits. Our study may provide novel knowledge for exploring the population structure and genetic potential of HT, offering a theoretical basis for its breeding strategies in the future.

## 1. Introduction

The Huitang duck (HT) is an indigenous breed that has been domesticated for an extensive period in Ningxiang (28°01′ N 112°22′ E), Hunan Province, China. According to local chronicles, the origin of HT can be traced back to the Ming dynasty (1368 AD–1644 AD). Over the years, in Hunan, various ducks have been selectively bred to meet the demand for meat and eggs, with the Huitang duck also being a dual-purpose type of breed. Several studies have indicated that a free-range raising system and outdoor access enhanced welfare in bird, leading to a substantial improvement in the quality and flavor of poultry meat and meat products [1,2,3]. Long-term domestication in hot spring areas and rice fields has provided an ideal environment for HT with superior animal welfare and may contribute to the unique meat flavor and breed characters [4].

In recent years, there have been many studies reporting on some indigenous famous duck breeds. The Linwu duck (LW, Hunan Province), known as a versatile breed for meat, boasts excellent meat quality and unique meat taste and texture [5,6]. The Youxian sheldrake duck (YX, Hunan Province), a versatile breed popular for its high egg-production rate and excellent egg quality [7,8]. The Jinding duck (JD, Fujian Province) is popular for its high laying performance [9,10]. A study on artificial selection between JD and meat-type ducks found that the candidate genes of JD ducks are mainly enriched in embryonic development function and metabolic pathways [11]. A population-level genomic analysis between the Korean-native duck and YX identified the genomic characteristics of meat flavor and texture phenotypes [12]. Jiang et al. [13] conducted a comparison between Chinese and South Asian ducks, revealing the prevalent gene flow occurrences across different regions. A comparative analysis involving typical breeds such as JD was carried out, promoting the preservation of duck diversity in China [14]. The analysis of population structure and genetic characteristics of native duck breeds by genome sequencing technology is a useful strategy to understand the genetic potential and breeding program of any novel duck breed [15].

However, as a vital component of Chinese local duck genetic resources, HT has not been fully explored in the existing literature. Here, we measured the phenotypic traits of HT and performed principal component, phylogenetic tree, structure, and LD decay analyses among HT and various duck types, including egg, meat, dual-purpose, and wild ducks, revealing that HT is a dual-purpose breed. Additionally, we conducted a selective sweep analysis among HT, LW, JD, YX, and mallard (MDN) ducks, revealing key candidate genes potentially associated with muscle development (*PLCG2*, *FN1*, *IGF2BP2*, *IGF1R*, and *IGFBP5*), follicle ovarian development (*GRIK4*, *MAP3K8*, *TGIF1*, *FOXO3*, and *ITPR1*), and egg-laying behaviors (*ERBB4*) in HT. This may provide valuable insights for future breeding schemes and genetic preservation of HT.

## 2. Materials and Methods

### 2.1. Materials

The HT in this study were reared in a cage-free semi-aquatic environment on a local farm, and supplementary feeding was provided (Table S1, Supplementary methods). We randomly selected 40 HT (90 days; 20 ducks of each sex) with similar body weight and health conditions for slaughter and evaluation. From these, three blood samples were collected from these birds for DNA extraction. Blood samples from three other breeds (LW, YX, and Sichuan sheldrake (SC)) were also collected from materials already in the laboratory. Moreover, six ducks were randomly selected to determine their meat quality and amino acid content. Eggs (green-shelled: 20, white-shelled: 20) of HT were randomly collected at 140 days–180 days to determine the quality and measure the amino acid content in 20 of them. The experimental procedures described above were approved by the Animal Ethics Supervisory Committee of Hunan Agricultural University and were performed in accordance with Animal Welfare China guidelines.

### 2.2. Methods

#### 2.2.1. Traits Measurements

The body size and slaughter performance of HT were measured. The quality of HT meat was determined based on parameters such as muscle colorimetric parameters (L*, a*, b*), pH (45 min), pH (24 h), and shear force (N). The egg quality of HT was determined by parameters including egg weight, shape index, egg shell thickness, egg shell strength, Haugh unit, albumen height, yolk color, yolk weight, and yolk ratio. In addition, we determined the amino acid composition of HT meat and eggs. The details of the experimental and calculation methods can be found in the Supplementary Methods. The differences between male and female were analyzed by a Student’s *t*-test with SPSS 16.0 (SPSS, Chicago, IL, USA), and the significance threshold was set at *p <* 0.05.

#### 2.2.2. Whole-Genome Resequencing

DNA was extracted from whole blood samples of three Hunan local duck breeds (HT, YX, and LW; n = 3) and SC (n = 3) using a Rapid Blood Genomic DNA Isolation Kit (Sangon Biotech, Shanghai, China). The DNA samples were sequenced by Huazhi Biotech Co., Ltd. (Changsha, China). Additionally, a total of 92 ducks’ genomic data, including representative wild breeds (Ningxia mallard (MDN), Zhejiang mallard (MDZ), and spot-billed ducks (SB)), meat breeds (Pekin (PK), Cherry Valley (CV), and maple leaf (ML)), egg breeds (Jinding duck (JD), Shanma duck (SM), and Shaoxing duck (SX)), and dual-purpose breeds (Sansui duck (SS), Taiwan sheldrake (TW), Gaoyou duck (GY), and Mei duck (M)) were downloaded from the NCBI database (https://www.ncbi.nlm.nih.gov/ accessed on 31 October 2022), and detailed information is provided in Table S2.

#### 2.2.3. Data Pre-Processing

All raw data from 104 ducks were processed using a stringent and uniform pipeline as follows. Briefly, we first removed adaptors and low-quality reads from the raw data using Trimmomatic (v0.39) [16]. Reads were aligned to the reference genome (CAU_Pekin_2.0) [17] using BWA (v0.7.17) [18], and hard filtering was performed using GATK (v4.1.9) [19] with the following parameters: QD < 2.0, FS > 60.0, MQ < 40.0, MQRankSum < −12.5, and ReadPosRankSum < −8.0. Variants were filtered both per population and per individual using VCFtools (v0.1.14) [20] with settings: “--maf 0.05 --max-missing 0.9 --hwe 0.000001 --min-alleles 2 --max-alleles 2”. Finally, SNPs were imputed using Beagle 5.4 [21], and their annotation was implemented via SnpEff (v4.3) [22].

#### 2.2.4. Population Structure Analysis

The population structure of HTw was analyzed by performing principal component analysis (PCA) using PLINK (v1.90) [23] with “-pca” command across 17 breeds of ducks. We constructed a neighbor-joining (NJ) tree from SNPs using VCF2Dis-1.47 (https://github.com/BGI-shenzhen/VCF2Dis accessed on 10 March 2022), which was visualized by iTOL (v6.7.4) [24]. Maximum likelihood estimates of population assignments for each individual were obtained with ADMIXTURE (v1.30) [25] (K = 2–5). The correlation coefficient (r^2^) between alleles at pairs of SNPs was calculated using PopLDdecay (v3.42) [26] to estimate the level of linkage disequilibrium (LD) in six duck populations: PK, JD, LW, YX, HT, and MDN. The objective was to understand the differences in LD decay patterns between different types of ducks and geographically proximate ducks compared to HT.

#### 2.2.5. Selective-Sweep Analysis

HT, LW, and JD were compared with MDN to detect candidate divergent regions (CDRs). We utilized VCFtools (v0.1.14) to calculate the fixation index (*F*_ST_) for single SNP and Tajima’s D using “--TajimaD 5000”. Using selscan (v2.0) [27], we computed and normalized the integrated haplotype score (iHS) and cross-population extended haplotype homozygosity (XP-EHH). We considered the intersection of the top 1% (right tail) of *F*_ST_, XP-EHH and the bottom 1% (left tail) of Tajima’s D and iHS as putative CDRs. The flanking region size of the CDRs was defined as the genomic distance at which LD decayed to the baseline in HT (100 Kb). Genes within the ±100 kb flanking regions of the overlapping CDRs between HT and LW as well as between HT and JD were identified as candidate genes. Furthermore, we calculated four statistics (*F*_ST_, Tajima’s D, iHS, and XP-EHH), as mentioned above, for HT and YX to discern genomic divergence. The subsequent visualization was conducted by the R package CMplot (v4.3.1) [28].

#### 2.2.6. Functional Enrichment Analysis

Metascape (v3.5) [29] and KOBAS (v3.0) [30] were used to analyze the functional annotations of candidate genes mentioned above by aligning with the GO (Gene Ontology) database (http://geneontology.org/ accessed on 4 March 2024) and the KEGG (Kyoto Encyclopedia of Genes and Genomes: https://www.genome.jp/kegg/ accessed on 4 March 2024) database, with a threshold of *p <* 0.05.

## 3. Results

### 3.1. Summary of HT Traits

**Figure 1:**
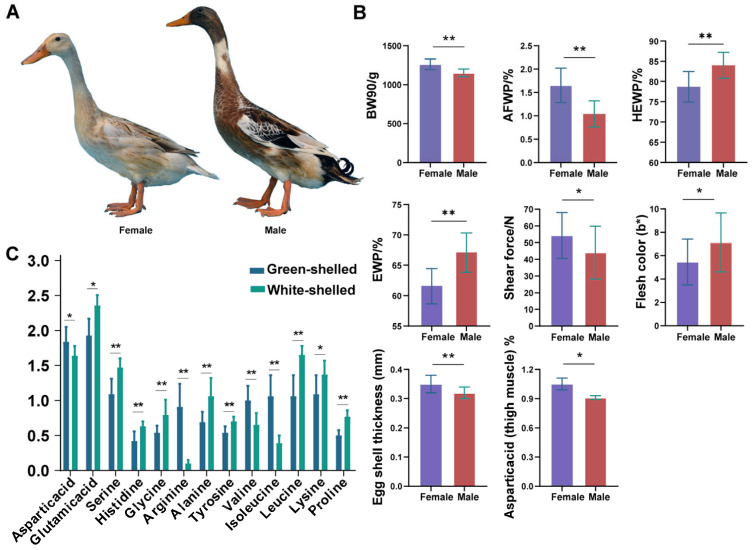
The appearance of HT and comparative analysis of phenotypes between males and females. (**A**) Appearance of female and male HT. (**B**) Traits of HT ducks (BW90, 90-day body weight; AFWP, abdominal fat weight percentage; HEWP, percentage of half-eviscerated yield; EWP, eviscerated weight percentage). (**C**) Amino acid content in green- and white-shelled eggs. * *p ≤* 0.05; ** *p ≤* 0.01.

The phenotypic traits, including body size and carcass traits, of 20 male HT were compared with those of 20 females (Figure 1A). Parameters showing significant differences between males and females of HT are presented in Figure 1B. The 90-day body weight (BW90) (*p <* 0.01) and abdominal fat weight percentage (AFWP) (*p <* 0.01) of females were significantly greater than those of males, while the percentage of half-eviscerated yield (HEWP) (*p <* 0.01) and eviscerated weight percentage (EWP) (*p <* 0.01) of females were markedly lower than those of males. However, for the other measurement parameters—body size, dressing percentage, breast muscle weight percentage, and thigh muscle weight percentage—differences between females and males existed but were not significant (*p >* 0.05) (Table S3).

The quality of muscle and eggs in HT was also determined (Table S4). The effects of sex on the b* value of thigh muscle and the shear force of breast muscle were significant (*p <* 0.05), which are shown in Figure 1B. Green-shelled eggs exhibited significantly thicker eggshells (*p <* 0.01) compared to white-shelled eggs (Figure 1B). However, no significant differences were observed in egg weight, egg shape index, eggshell strength, Haugh units, albumen height, yolk color, yolk weight, and yolk ratio between green-shelled and white-shelled eggs (*p >* 0.05) (Table S5).

The amino acid composition of breast muscle, thigh muscle for male and female ducks, as well as eggs (green-shelled and white-shelled), is detailed in Table S6. No significant differences were found in the contents of glutamic acid (Glu), serine (Ser), histidine (His), glycine (Gly), threonine (Thr), arginine (Arg), alanine (Ala), tyrosine (Tyr), valine (Val), methionine (Met), phenylalanine (Phe), isoleucine (Ile), leucine (Leu), and proline (Pro) between male and female ducks’ breast and thigh muscles (*p >* 0.05). However, compared to female ducks, males exhibited higher concentrations of aspartic acid (Asp) in thigh muscles. Additionally, compared to green-shelled eggs, white-shelled eggs showed sharply higher concentrations of Ser, His, Gly, Ala, Tyr, Leu, and Pro (*p <* 0.01) and Lys and Glu (*p <* 0.05), while concentrations of Arg, Val, and Ile (*p <* 0.01) and Asp (*p <* 0.05) were significantly lower (Figure 1C).

### 3.2. Population Structure Analysis

**Figure 2:**
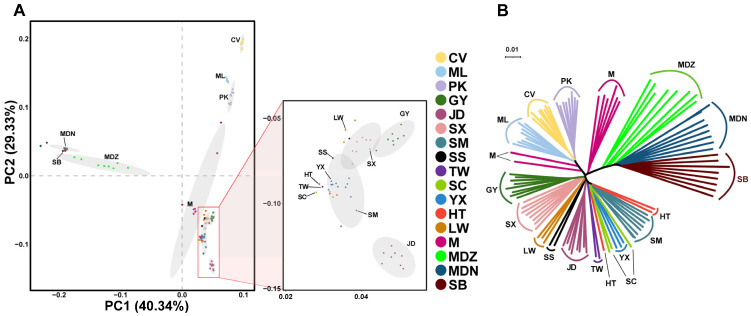
Population structure analysis of 17 duck breeds (CV, Cherry Valley; ML, maple leaf; PK, Pekin; GY, Gaoyou duck; JD, Jinding duck; SX, Shaoxing duck; SM, Shanma duck; SS, Sansui duck; TW, Taiwan sheldrake; SC, Sichuan sheldrake; YX, Youxian sheldrake; HT, Huitang duck; LW, Linwu duck; M, Mei duck; MDN, Ningxia mallard; MDZ, Zhejiang mallard; SB, Chinese spot-billed duck). (**A**) Principal component analysis of the duck samples. Principal components 1 (40.34%) and 2 (29.33%) explained the variability among the 104 ducks. (**B**) Neighbor-joining phylogenetic tree analysis of 17 duck populations.

In this study, a total of 466 Gb of data was downloaded from NCBI database, involving 92 individuals from 16 different breeds. We generated 74.3 Gb whole-genome sequencing data (5×) for 12 ducks from HT, LW, YX, and SC with 150 bp paired-end reads. After quality control, a set of 11,631,231 high-quality SNPs were obtained, which were used to characterize genetic relationships between HT and 16 other duck breeds with PCA and a neighbor-joining method. The PCA results showed that 17 duck breeds were divided into three major clusters (Figure 2A). The wild ducks (MDZ, MDN, and SB) were roughly clustered together, as were the meat duck breeds (CV, ML, and PK). Among the remaining samples, the dual-purpose breeds (HT, LW, YX, SC, TW, SS, GY, and M) were tightly clustered together alongside egg duck breeds (JD, SM, and SX). M appeared to be an outlier, located between wild and meat duck breeds. Consistent with the PCA, the NJ tree of pairwise genetic distances showed that egg ducks (JD, SM, and SX) clustered within one branch next to the dual-purpose breeds, while wild ducks and meat ducks clustered in distinct branch. This suggests that most individuals within the same breed share a similar genetic structure (Figure 2B).

**Figure 3:**
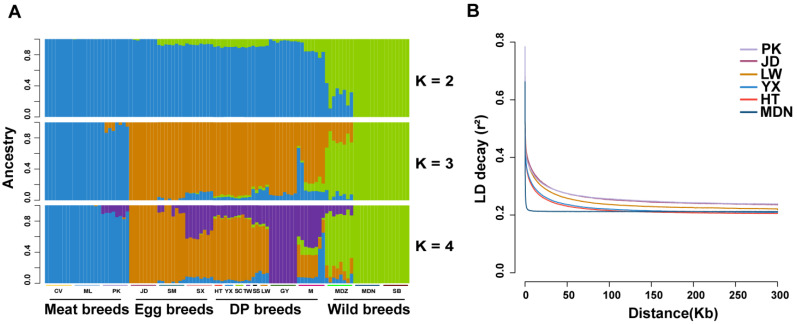
Population ADMIXTURE and LD decay analysis. (**A**) Population genetic structure of 104 ducks, where the length of each colored segment represents the proportion of the individual’s genome inferred from ancestral populations (K = 2–4). The population names and production types are listed at the bottom (DP, dual-purpose). (**B**) Genome-wide linkage disequilibrium analysis of ducks (PK, JD LW, YX, HT, and MDN).

The ADMIXTURE result revealed the potential population genetic exchanges. When K = 2, a clear division was found between wild ducks (MDZ, MDN, and SB) and the rest of the duck breeds (Figure 3A). When K = 3, a distinction emerged between ducks reared primarily for meat production (PK, CV, and ML) and a combination of egg and dual-purpose ducks (JD, SM, SX, and GY), featuring the lowest CV (cross-validation) error (Figure S1). Among these, JD appeared as a pure breed without admixture from any meat or wild breeds. When K = 4, LW showed more ancestry admixture from meat breeds than HT and YX. Additionally, HT, YX, SC, and TW displayed similar population admixture patterns. We then calculated the pairwise correlation coefficient of the SNPs for MDN, HT, LW, YX, PK, and JD to investigate the selective pressure exerted on the population. As expected, LD analysis showed that MDN, as the wild duck breed, had the fastest LD decay rate, while HT genomes exhibited relatively short LD distances and a faster decay of the pairwise correlation coefficient compared to PK, JD, and LW, indicating that HT is less domesticated (Figure 3B).

### 3.3. Genome-Wide Selective Sweep Analysis

**Figure 4:**
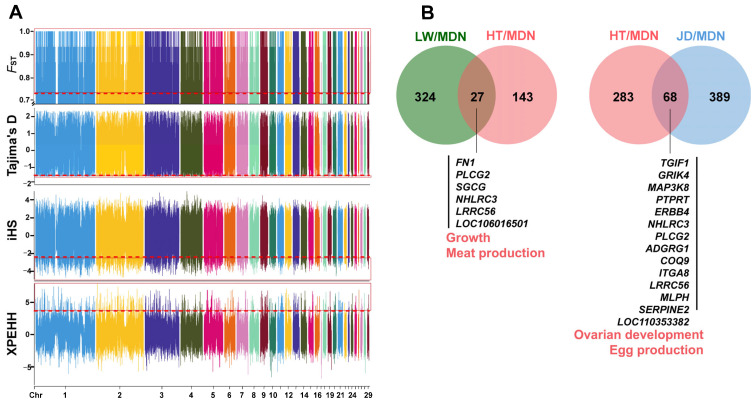
Genomic regions with strong selective signals in ducks. (**A**) Distribution of *F*_ST_, Tajima’s D, iHS, and XPEHH; the *x*-axis represents the chromosomes. The *F*_ST_ and XP-EHH were calculated for a single SNP between HT and MDN. Tajima’s D (5-kb window) and iHS (single SNP) were calculated for HT. The 1% of these statistics is considered indicative of selection in HT, with the thresholds set at *F*_ST_ > 0.77, Tajima’s D < −1.49, iHS < −2.41, and XPEHH > 3.37. The red dashed line represents the threshold for the statistical measure, while the red box delineates the 1% distribution range of the statistical measure. (**B**) Venn diagram depicting the number of unique and overlapping CDRs from the top 1% of *F*_ST_, Tajima’s D, iHS, and XPEHH. Numbers represent the counts of CDRs in each group, along with annotations of genes related to meat and egg production in overlapping CDRs identified by the four statistics.

To measure population divergence, we compared HT, LW, and JD with mallards (MDN), respectively, for selective signal analysis. The genome was scanned for regions exhibiting extreme distributions of *F*_ST_, Tajima’s D, iHS, and XP-EHH to identify candidate divergent regions (CDRs) on autosomes. Using a 1% cutoff (*F*_ST_ > 0.77, Tajima’s D < −1.50, iHS < −2.41, and XP-EHH > 3.37), we identified 351 CDRs under selection in HT compared with MDN (Figure 4A). A similar analysis conducted for LW versus MDN and JD versus MDN yielded 170 and 457 CDRs, respectively. Detailed threshold information is provided in Table S7. We retrieved these CDRs between HT, LW, and JD and considered the overlapping regions to be under positive selection during both natural and artificial selection.

**Figure 5:**
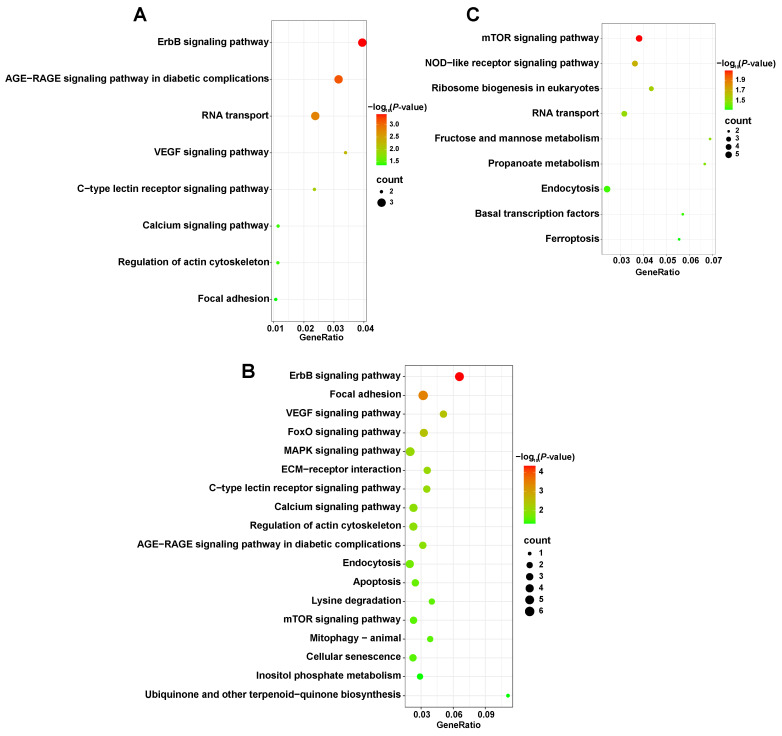
Enrichment analysis of KEGG pathways. (**A**) Analysis of shared CDRs between HT and LW. (**B**) Analysis of shared CDRs between HT and JD. (**C**) Analysis of shared CDRs between HT and YX.

There are only 27 overlapping CDRs between HT and LW (Figure 4B), encompassing 74 candidate genes functionally involved in RNA localization (GO:0006403), regulation of cellular component size (GO:0032535), and the Wnt signaling pathway (GO:0016055) (Figure S2A). We identified phospholipase C gamma 2 (*PLCG2*) on chromosome 12, which is associated with the Wnt signaling pathway (GO:0016055). Fibronectin 1 (*FN1*) was involved in the regulation of cellular component size (GO:0032535). In addition, erb-b2 receptor tyrosine kinase 4 (*ERBB4*) is associated with telencephalon development (GO:0021537), and the insulin like growth factor 2 mRNA binding protein 2 (*IGF2BP2*) gene is related to RNA localization (GO:0006403). It is interesting to find that *PLCG2*, *FN1*, and *ERBB4* are involved in multiple signal transduction pathways of KEGG, such as the calcium signaling pathway, the regulation of actin cytoskeleton, and focal adhesion (Figure 5A).

Moreover, we retrieved 68 overlapping CDRs for HT and JD, which contain 213 candidate genes (Figure 4B). A total of twenty GO terms were enriched by these genes, including postsynaptic density (GO:0014069), regulation of Ras protein signal transduction (GO:0046578), regulation of MAPK cascade (GO:0043408), and others (Figure S2B). It is worth noting that glutamate ionotropic receptor kainate type subunit 4 (*GRIK4*) participated in processes related to synapses and metal ion transport (GO:0051966, GO:0014069, GO:0098793, and GO:0046873). *ERBB4* and *PLCG2* were both involved in synaptic processes and the regulation of the MAPK cascade (GO:0014069, GO:0043083, and GO:0043408). Additionally, the candidate genes mentioned above are primarily linked to 18 KEGG pathways, including the MAPK signaling pathway, which contains mitogen-activated protein kinase kinase kinase 8 (*MAP3K8*) and *ERBB4* (Figure 5B).

### 3.4. Detection of Candidate Divergent Regions between HT and YX

**Figure 6:**
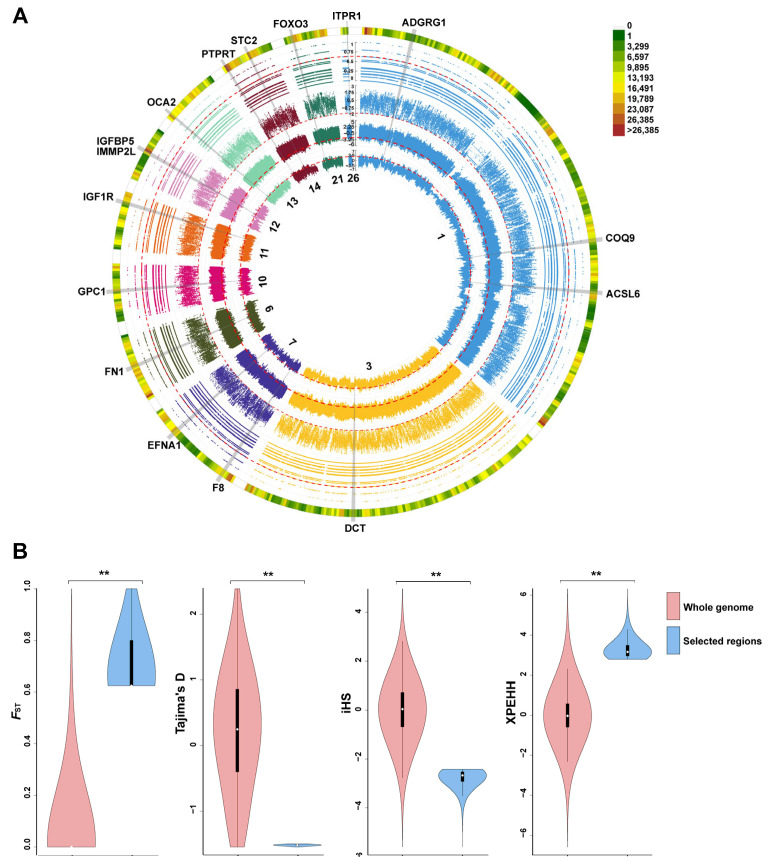
The selective sweep analysis between HT and YX. (**A**) From outer to inner, the outermost circle represents SNP density on chromosomes, the second circle represents *F*_ST_, the third circle represents Tajima’s D, the fourth circle represents iHS, and the innermost circle represents XP-EHH. Genes associated with important economic traits in HT are marked. (**B**) Violin plot of *F*_ST_, Tajima’s D, iHS, and XP-EHH for duck genomic regions that have undergone strong selection, compared to the whole genome. The statistical significance was calculated using the Mann–Whitney U test. ** *p* < 2.2 × 10^−16^.

In total, we identified 9.3 Mb regions that exhibited significant differences (*p <* 2.2 × 10^−16^, Mann–Whitney U test) in selective sweeps: *F*_ST_, Tajima’s D, iHS, and XP-EHH values compared to the whole genome between HT and YX, representing 0.77% of the HT genome (Figure 6A). We pinpointed 93 CDRs containing 392 genes, situated within the extreme top 1% of the statistical distribution (Figure 6B). Among these candidate genes, insulin-like growth factor 1 receptor (*IGF1R*), forkhead box O3 (*FOXO3*), insulin like growth factor binding protein 5 (*IGFBP5*), and inositol 1,4,5-trisphosphate receptor type 1 (*ITPR1*) were related to growth factor binding (GO:0019838), cell population proliferation (GO:0008283), cellular response to extracellular stimulus (GO:0031668), and channel activity (GO:0015267) (Figure S2C). *IGF1R* and *ITPR1* were, respectively, enriched in the mTOR signaling pathway and NOD-like receptor signaling pathway, which were within the top nine pathways (*p <* 0.05) (Figure 5C).

## 4. Discussion

Growth and development serve as key criteria in poultry breeding and selection, with body size reflecting developmental status, particularly the growth of muscles and bones [31]. Internal quality traits of eggs play a crucial role in the context of poultry egg production [32]. The flavor and nutritional value of duck meat are directly influenced by the composition and quantity of amino acids; a higher content of amino acids leads to a sweet and meaty aroma [33,34]. For example, glutamate can impart a pleasant, fresh taste [35]. In this study, we systematically measured important phenotypic traits of HT, characterizing its slaughter performance and meat and egg quality.

To investigate the population genetic structure of HT, we conducted a comparative analysis between HT and 16 categorized duck breeds based on their genetic background elucidated in prior studies [11,13]. The breeding of Chinese domestic ducks dates back to the Ming dynasty (AD 1367) [36]. The significant geographic separation over an extended period and the selection imposed for different production purposes led to differentiation of wild, meat, and egg ducks breeds into distinct groups [36]. Notably, HT, LW, and YX clustered together and were positioned next to the egg duck breeds, which, associated with domestic ducks along the Yangtze-Huai region, exhibit considerable connectedness and gene flow [36]. When K = 4, among the local duck breeds in Hunan, LW showed more ancestry admixture from meat breeds than HT and YX in the ADMIXTURE results (Figure 3). This could be attributed to the local preference for breeding LW for its meat-producing traits [37], leading to genomic differentiation between LW and the other two breeds in Hunan, namely HT and YX. Additionally, HT, YX, and SC exhibited similar population genetic structures. Historical records indicated a large-scale human migration from Hunan to Sichuan during the Qing dynasty in AD 1714, which may have resulted in the lineage of Hunan-native duck breeds flowing into SC. Overall, the results of the population structure analysis consistently indicate that HT is a dual-purpose breed with a similar genetic background to YX.

To understand the selection pressures on meat production and egg-laying traits in HT, we identified the overlapping CDRs between domestic ducks (meat duck: LW; egg duck: JD) and HT. These CDRs contain 74 genes, including *PLCG2*, which is implicated in the calcium signaling pathway, and *FN1*, involved in the regulation of the actin cytoskeleton and focal adhesion. Cao et al. [38] found that the calcium signaling pathway is associated with muscle development in the Pekin duck and Hanzhong Ma duck. The regulation of the actin cytoskeleton participates in regulating the development of skeletal muscle in the Pekin duck during the embryonic stage [39]. Chen et al. [40] reported that focal adhesion is related to growth of skeletal muscle in black Muscovy duck. Hu et al. [41] found that *FN1* is involved in the growth and development of duck skeletal muscle. *IGF2BP2* plays an important role in metabolism and may be associated with muscle development and growth performance during early growth stages in poultry [42]. Therefore, based on our results, *PLCG2, FN1,* and *IGF2BP2* were enriched in these signaling pathways related to muscle growth and development and may be important candidate genes affecting meat production in HT.

We screened some genes (e.g., *GRIK4*, *MAP3K8*, *TGIF1*, and *ERBB4*) within the shared CDRs between HT and JD, which are involved in neuroactive ligand–receptor interaction, MAPK signaling pathway, calcium signaling pathway, and TGF-beta signaling pathway. Lin et al. [38] found that the MAPK signaling pathway affects follicular development and ovulation of Muscovy duck. The calcium signaling pathway has an important effect on duck reproductive behavior [43]. *TGIF1* is a member of the TALE homeodomain protein family, involved in various physiological processes, including lipid and carbohydrate metabolism and the inhibition of androgen receptor activity [44]. *ERBB4* participates in modulating the MAPK signaling pathway, influencing the egg-laying behavior of poultry [45]. Chen et al. [46] reported that the TGF-beta signaling pathway is essential for ovarian development. *GRIK4* is a candidate gene affecting age-at-first-egg traits in Shaoxing ducks [47]. In recent study, *MAP3K8* was identified as a candidate gene for egg production of Muscovy ducks [48]. Additionally, some genes, such as *GRIK4* and *MAP3K8*, were identified to exhibit positive selection sweeps in the genomes of HT and JD, indicating that during the domestication process of HT, certain regions of the genome may experience selection in the same direction as JD.

The comparation between HT and YX identified several candidate regions covering highly differentiated genes. Among these, *IGF1R*, *ITPR1,* and *FOXO3* were found to be involved in the mTOR signaling pathway, NOD-like receptor signaling pathway, calcium signaling pathway, and FoxO signaling pathway. Some studies have proposed that the mTOR signaling pathway affects myoblast proliferation and breast muscle yield [49,50]. Recent studies have shown that the NOD-like receptor signaling pathway is essential for ovarian development in poultry [46,51]. The FoxO signaling pathway plays a regulatory role in chicken granulosa cell function and affects follicular development and ovulation in ducks [52,53]. Additionally, it was reported that *IGF1R* serves as a candidate marker for growth and skeletal development in ducks [15]. *IGFBP5* was detected in the transcriptomic profile, potentially influencing leg muscle development during early growth in chickens [42]. Liu et al. [54]. found that *ITPR1* influences egg-production performance through the calcium signaling pathway. In our study, *IGFBP5* and *IGF1R*, which might influence muscle development, were identified in the selective sweep analysis of HT and YX. Additionally, *ITPR1* and *FOXO3*, associated with signaling pathways related to follicular and ovarian development, were also detected. Therefore, we consider these genes to be noteworthy in the selection and breeding of crucial candidate genes for important economic traits in HT.

## 5. Conclusions

In this study, for the first time, we investigated the phenotypic and genetic background of HT and comprehensively compared selective signatures on HT genome to other duck breeds. The population structure analysis revealed that HT is a dual-purpose breed, sharing similar bloodlines with YX and distinguished from other duck breeds. Through selection signal analysis, we identified 95 selective sweeps, covering genes including three (*PLCG2*, *FN1,* and *IGF2BP2*) in HT that are potentially related to growth traits and four (including *GRIK4*, *MAP3K8*, *TGIF1*, and *ERBB4*) that may be involved in regulating egg production. The genomic comparison between HT and YX revealed 93 selective sweeps, where four crucial genes associated with meat production (*IGF1R* and *IGFBP5*) and egg production (*FOXO3* and *ITPR1*) were identified. Our study may provide novel insights into the population structure and genetic potential of HT, offering a theoretical basis for its future breeding objectives.

## Data Availability

The dataset supporting the conclusions of this article is available with links to BioProject accession number PRJNA1088559 (https://www.ncbi.nlm.nih.gov/bioproject/PRJNA862275, accessed on 1 October 2024).

## Supplementary Materials

The following supporting information can be downloaded at: https://www.mdpi.com/article/10.3390/ani14121747/s1, Supplementary Methods: Integrated rice-duck farming, body size, carcass characteristics, meat quality, amino acid composition of muscle and eggs, and egg shell quality measurement; Figure S1: The cross-validation (CV) error of ADMIXTURE analysis; Figure S2: Enrichment analysis of GO terms; Table S1: Nutrient levels of the experimental diets; Table S2: The basic and genomic information of 92 ducks; Table S3: Descriptive statistics of body size and carcass traits of 90-day HT; Table S4: Meat quality of the HT; Table S5: Egg quality measurements of HT; Table S6: Amino acid content in HT meant and eggs; Table S7: The threshold information of HT selective sweep analyses.
