# Supplementary material for: Phenotypic Identification, Genetic Characterization, and Selective Signal Detection of Huitang Duck
Source: Animals (Basel). 2024 Jun 10;14(12):1747. doi: 10.3390/ani14121747 (PMC11201145; doi:10.3390/ani14121747)
Supplement: Supplementary file 1 [file animals-14-01747-s001.zip › animals-2941929-supplementary.pdf]

Supplementary Materials for

Ma, et al. Phenotypic Identification, Genetic Characterization, and Selective Signal Detection of Huitang Duck

CONTENTS

Supplementary methods (Page 2-3)

Supplementary Figures (Page 4)

Supplementary Tables (Page 5-9)

Supplementary methods

Integrated rice-duck farming

Before the experiment, the paddy fields were thoroughly fertilised (before transplanting). No pesticides, fertilizers or herbicides were applied to the rice during the experiment. After rice transplantation, 20-day-old ducklings (after completion of all immunisation procedures) were introduced into the paddy fields at a density of about 18 ducklings per 660 m^2. They were fed twice a day. The water depth was initially 5 cm and gradually increased to 8-10 cm over a transition period of 3-5 days. The co-culture of rice and ducks was terminated when the rice reached the heading and grain filling stages.

Body Size Measurements

The body dimensions were body length (BL), length between the first cervical vertebra and the pygostyle; breast width (BrW), distance between left and right glenoid cavity; breast depth (BD) was measured from the first back vertebra to the sternum; keel bone length (KBL), distance from anterior to posterior end of the keel; pelvis width (PW), distance between two hip joints; shank length (SL) was measured from the shank joint to the extremity of the digits pedis; shank circumference (SC), distance around the middle part of the shank or humerus; Half-diving length (HDL), distance from the tip of the mouth to the midpoint of the hip joint. Tape measure and Vernier caliper were used to measure the body sizes in centimeters at a precision of 0.1 and 0.01 cm respectively. The data of the body sizes of the first generation were used to draw the body size growth curve of HT.

Carcass Characteristics

We randomly selected 40 HT (90d, 20 ducks of each gender) with similar body weight and health conditions. Following an 8-hour period of fasting, weighed live weight (BW90) of ducks. The weight of ducks after plucking and bloodletting was taken as dressed weight (DW) The carcass was then manually eviscerated, weighed after removing viscera including the crop, trachea, esophagus, spleen, pancreas, gallbladder, gonads, and intestinal tract, which was recorded as half-eviscerated weight (HEW). After removal of head, foot, and viscera was taken as eviscerated weight (EW). Dressed percentages was calculated by DW/BW. Eviscerated yield was calculated as the percentages of BW. Breast muscle, thigh muscle, and abdominal fat pad including leaf fat surrounding the cloaca and gizzard were separated and weighed. Breast and thigh muscle yields were calculated as the percentages of EW. Abdominal fat percentage was calculated by abdominal fat weight/(abdominal fat weight + EW). Subsequently, within 10 min postmortem, all the right entire pectoralis majors and thigh muscle were collected for the determination of meat quality.

Meat Quality

Color, pH and shear force of the meat were all assessed in the right breast and thigh muscles. L* (lightness), a* (redness), and b* (yellowness) of three random locations surface of the duck breast and thigh meat were measured using a colorimeter (Konica Minolta Sensing Inc., Osaka, Japan) 1 h postmortem. At 45 min and 24 h after slaughtering, the pH of breast and thigh muscles were measured with a pH meter (pH-STAR, SFK technology, Denmark), previously calibrated with pH 4.6 and 7.0 buffers. Using a digital tenderness meter (C-LM3B, Tenovo Food, Beijing,

China), muscle strips with fibers perpendicular to the blade were sheared to determine shear force.

Amino Acid Composition of Muscle and Eggs

About 150 mg breast and thigh muscle were weighed into a glass bottle and 15 mL of 6 mol HCl were added. After nitrogen filling, the mixture was hydrolyzed for 22–24 h at 110 ◦C. Next, the hydrolysate was transferred to a 50 mL volumetric flask and diluted to calibration tail with ultrapure water. The solution was filtered using a 0.45 μm membrane filter into an autosampler vial, and then analyzed by L-8900 amino acid analyzer (HITACHI, Japan). Egg solution (5 mL) was mixed with 5 mL of 16% trichloroacetic acid solution (FUJIFILM Wako Chemicals, Japan). After vertexing, the samples were centrifuged at 1,400 g for 15 min using a table-top centrifuge, model 2410 (KUBOTA Corporation Co., Ltd., Japan). The supernatant was collected using a 5 mL syringe (NIPRO Corporation, Japan) and filtered through a disposable cellulose acetate membrane filter unit with a 0.45 μm pore size (DISMIC-25CS; Advantec Toyo Kaisha, Ltd., Japan). After heating at 40°C for 60 min in a vacuum oven (VOS-201SD, Eyela, Japan), 20 mL of mixing solution (ethanol: DW: TEA = 2:2:1) was added to the tube and then mixed for 20 min using a micro tube mixer MT-360 (Tomy Seiko Co. Ltd., Japan). The sample was heated at 40°C for 60 min in a vacuum to dry. After adding 20 mL of mixing solution (Ethanol: DW: TEA: PITC = 7:1:1:1) and mixing for 20 min, the sample was re-heated at 40°C for 60 min in a vacuum to dry. After preprocessing, the samples analyzed by L-8900 amino acid analyzer (HITACHI, Japan).

Egg Shell Quality

The egg shell quality traits were measured for 40 eggs (green shell: 20, white shell: 20) at 140d-180d. The following external traits were recorded for each egg: egg weight, egg length or vertical diameter, egg width or horizontal diameter, shape index which was defined as the ratio of the length on the width, egg shell breaking strength, egg shell thickness at the blunt region, equatorial region, and sharp region, and the average of these 3 thicknesses. Broken eggs were not used. Egg weight was measured to the nearest 0.01 g while the shell thickness was measured with a precision of 0.01 mm using a Vernier caliper. Breaking strength was measured by pole-to-pole pressing of the egg with a digital concrete compression testing machine with capacity range of 0 N to 500 N. The yolk color, albumen height, and Haugh unit of the duck eggs were measured using egg quality analyzer (EA-01, Israel ORKA Food Technology Ltd., Bountiful, UT).

Supplementary Figures

Figure S1. The cross-validation (CV) error (K=1-6) of ADMIXTURE analysis.

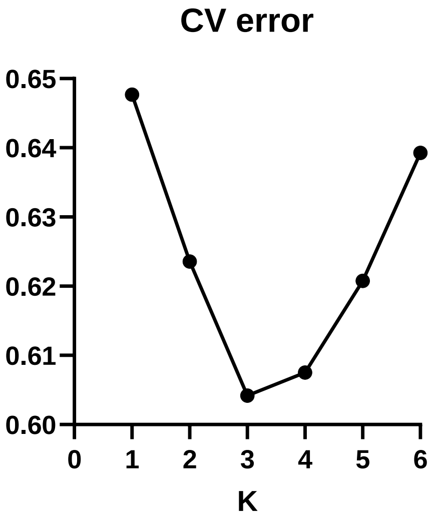

Figure S2. Enrichment analysis of GO terms. (A): Analysis of shared CDRs between HT and LW. (B): Analysis of shared CDRs between HT and JD. (C): Analysis of shared CDRs between HT and YX.

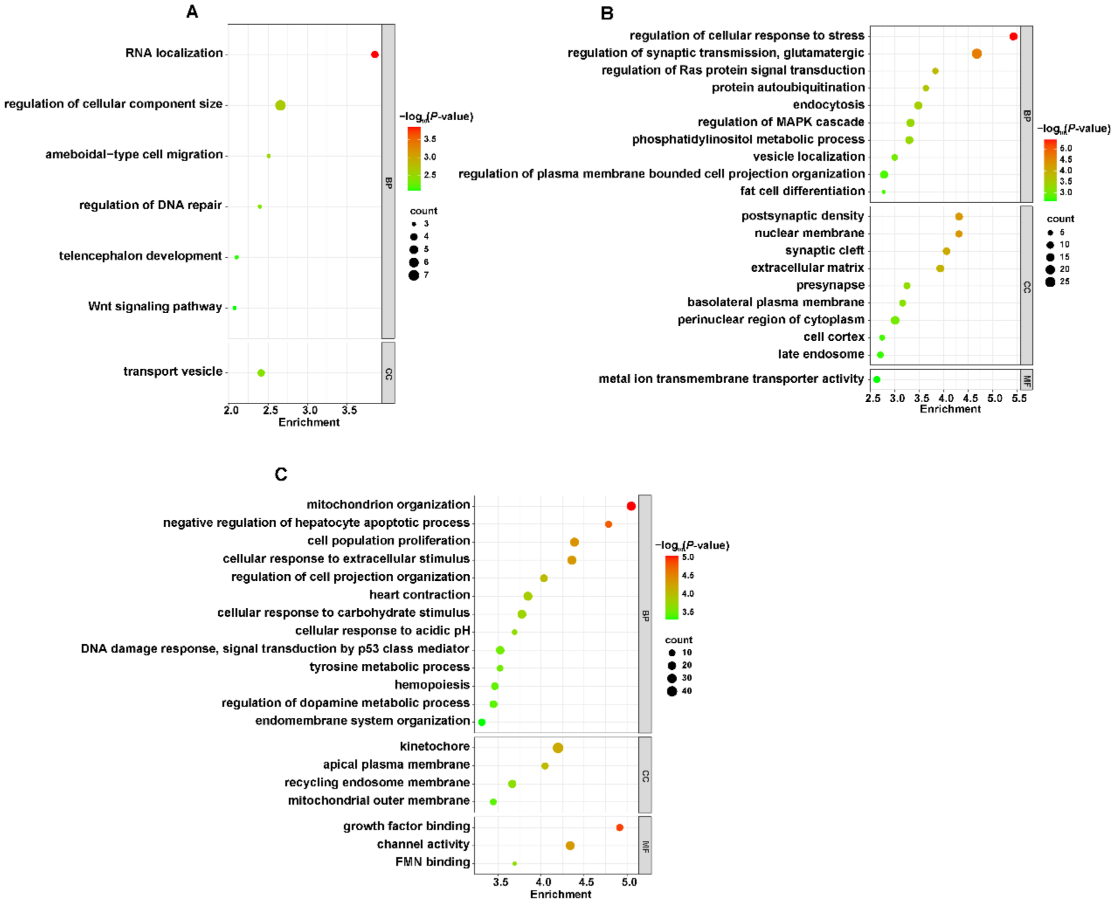

Supplementary Tables

Table S1. Nutrient levels of the experimental diets.

Item	content		
	1-3 weeks	4-7 weeks	8-13 weeks
Metabolizable energy (MJ/kg)	12.56	11.84	12.50
Crude protein (%)	19.31	17.56	15.64
Lysine (%)	1.05	0.85	0.65
Methionine (%)	0.40	0.38	0.35
Phosphorus (%)	0.65	0.60	0.55
Calcium (%)	0.90	0.80	0.80

Table S2. The basic and genomic information of 92 ducks.

Name	Abb.	Nmuber	BioProject	DOI.	Type
Mallard (Ningxia)	MDN	8	PRJNA419832	10.1093/gigascience/giy027	Wild breeds
Mallard (Zhejiang)	MDZ	8	PRJNA419832	10.1093/gigascience/giy027	Wild breeds
Spot-billed ducks	SB	8	PRJNA686828	10.1186/s12862-021-01894-7	Wild breeds
Pekin ducks	PK	8	PRJNA419832	10.1093/gigascience/giy027	Meat breeds
Cherry Valley ducks	CV	8	PRJNA419832	10.1093/gigascience/giy027	Meat breeds
Maple Leaf ducks	ML	8	PRJNA419832	10.1093/gigascience/giy027	Meat breeds
Jinding ducks	JD	8	PRJNA419832	10.1093/gigascience/giy027	Egg breeds
Shanma ducks	SM	8	PRJNA419832	10.1093/gigascience/giy027	Egg breeds
Shaoxing ducks	SX	8	PRJNA419832	10.1093/gigascience/giy027	Egg breeds
Sansui ducks	SS	2	PRJNA450892	10.1038/s41467-018-04868-4	Dual-purpose breeds
Taiwan sheldrake	TW	2	PRJNA450892	10.1038/s41467-018-04868-5	Dual-purpose breeds
Gaoyou ducks	GY	8	PRJNA419832	10.1093/gigascience/giy027	Dual-purpose breeds
Mei ducks	M	8	PRJNA686828	10.1186/s12862-021-01894-7	Dual-purpose breeds

Table S3. Descriptive statistics of body size and carcass traits of 90d HT.

**Item**	**Trait**	**Female (n = 20)**	**Male (n = 20)**	***P*-value**
Body size	Pelvis width (PW, mm)	70.54 ± 4.83	69.49 ± 6.09	0.55
	Breast width (BrW, mm)	72.64 ± 4.23	70.35 ± 3.14	0.06
	Breast depth (BD, mm)	73.82 ± 4.25	72.60 ± 4.44	0.38
	Shank length (SL, cm)	6.63 ± 0.78	6.86 ± 0.91	0.40
	Shank circumference (SC, cm)	3.94 ± 0.25	4.06 ± 0.45	0.30
	Body slope length (BSL, cm)	14.19 ± 1.51	13.37 ± 1.39	0.08
	Keel bone length (KBL, cm)	10.55 ± 1.73	10.35 ± 2.57	0.77
	Half-diving depth (HDP, cm)	44.55 ± 2.09	45.19 ± 2.16	0.35
Carcass yield	90-day body weight (BW90, g)	1260.90 ± 69.57	1151.25 ± 49.41	<0.01
	Dressing percentage (DP, %)	92.15 ± 2.63	91.25 ± 3.15	0.33
	Percentage of half-eviscerated yield (HEWP, %)	78.70 ± 3.79	84.00 ± 3.20	<0.01
	Eviscerated weight percentage (EWP, %)	61.56 ± 2.91	67.09 ± 3.23	<0.01
	Breast muscle weight percentage (BMWP, %)	10.74 ± 1.27	10.44 ± 1.41	0.48
	Thigh muscle weight percentage (LMWP, %)	12.81 ± 1.55	12.39 ± 1.90	0.45
	Abdominal fat weight percentage (AFWP, %)	1.65 ± 0.37	1.04 ± 0.28	<0.01

The statistical analysis between female and male groups.

Table S4. Meat quality of the HT.

**Item**		**Breast muscle**			**Thigh muscle**		
		Female (n = 6)	Male (n = 6)	*P*-value	Female (n = 6)	Male (n = 6)	*P*-value
	*L**	42.26 ± 5.94	43.88 ± 4.76	0.348	43.36 ± 4.79	45 ± 5.95	0.35
Flesh color	a*	14.98 ± 2.31	14.7 ± 2.14	0.694	16.16 ± 2.01	15.75 ± 2.80	0.61
	b*	4.79 ± 1.36	4.96 ± 1.33	0.700	5.45 ± 1.96	7.13 ± 2.52	0.03
pH (45 min)		6.2 ± 0.43	6.23 ± 0.21	0.751	6.26 ± 0.41	6.35 ± 0.23	0.43
pH (24 h)		6.16 ± 0.35	6.14 ± 0.28	0.842	6.24 ± 0.26	6.31 ± 0.23	0.38
Shear force (N)		54.23 ± 13.74	43.98 ± 15.82	0.035	27.2 ± 6.82	26.69 ± 7.93	0.83

Table S5. Egg quality measurements of HT.

**Item**	**Green-shelled eggs (n = 20)**	**White-shelled eggs (n = 20)**	***P*-value**
Egg weight (g)	54.10 ± 5.89	52.97 ± 5.79	0.54
Egg shape index	1.39 ± 0.06	1.38 ± 0.06	0.51
Egg shell thickness (mm)	0.35 ± 0.03	0.32 ± 0.02	<0.01
Egg shell strength (kg/cm^2)	3.78 ± 0.90	3.33 ± 0.97	0.13
Haugh unit	74.5 ± 11.88	69.94 ± 14.80	0.29
Albumen height (mm)	5.49 ± 1.73	5.03 ± 1.46	0.37
Yolk color	9.60 ± 1.23	9.70 ± 1.13	0.79
Yolk weight (g)	18.09 ± 2.40	17.69 ± 2.47	0.61
Yolk ratio (%)	33.48 ± 3.13	33.33 ± 1.59	0.84

The statistical analysis between green-shelled eggs and white-shelled eggs groups.

Table S6. Amino acid content in HT meant and eggs.

**Item**	**Breast muscle**			**Thigh muscle**			**Eggs**		
	Female (n = 6)	Male (n = 6)	*P*-value[1]	Female (n = 6)	Male (n = 6)	*P*-value[1]	green-shelled (n = 10)	white-shelled (n = 10)	*P*-value[2]
Aspartic acid	0.93 ± 0.03	0.88 ± 0.13	0.34	1.05 ± 0.06	0.91 ± 0.02	0.02	1.84 ± 0.21	1.64 ± 0.14	0.04
Glutamic acid	1.57 ± 0.07	1.54 ± 0.24	0.72	1.26 ± 0.18	1.29 ± 0.13	0.83	1.93 ± 0.24	2.36 ± 0.15	0.02
Serine	0.40 ± 0.02	0.38 ± 0.06	0.37	0.33 ± 0.03	0.35 ± 0.04	0.57	1.09 ± 0.22	1.47 ± 0.13	<0.01
Histidine	0.42 ± 0.05	0.38 ± 0.09	0.49	0.31 ± 0.10	0.35 ± 0.08	0.46	0.42 ± 0.14	0.63 ± 0.07	<0.01
Glycine	0.52 ± 0.03	0.52 ± 0.08	0.94	0.46 ± 0.09	0.50 ± 0.03	0.43	0.54 ± 0.10	0.79 ± 0.22	<0.01
Threonine	0.65 ± 0.02	0.63 ± 0.08	0.76	0.60 ± 0.09	0.55 ± 0.06	0.24	1.07 ± 0.12	1.21 ± 0.26	0.15
Arginine	0.89 ± 0.04	0.82 ± 0.13	0.33	0.66 ± 0.14	0.73 ± 0.07	0.32	0.91 ± 0.33	0.10 ± 0.05	<0.01
Alanine	0.72 ± 0.02	0.68 ± 0.10	0.61	0.55 ± 0.06	0.61 ± 0.05	0.06	0.69 ± 0.15	1.06 ± 0.26	<0.01
Tyrosine	0.29 ± 0.02	0.28 ± 0.05	0.73	0.30 ± 0.05	0.30 ± 0.05	0.98	0.54 ± 0.09	0.70 ± 0.07	<0.01
Valine	0.34 ± 0.01	0.32 ± 0.05	0.67	0.29 ± 0.13	0.37 ± 0.06	0.12	1.00 ± 0.21	0.65 ± 0.17	<0.01
Methionine	0.39 ± 0.01	0.40 ± 0.05	0.94	0.50 ± 0.21	0.55 ± 0.08	0.49	0.79 ± 0.17	0.95 ± 0.18	0.50
Phenylalanine	0.39 ± 0.01	0.36 ± 0.08	0.48	0.24 ± 0.04	0.24 ± 0.07	0.99	0.67 ± 0.05	0.52 ± 0.36	0.87
Isoleucine	0.30 ± 0.12	0.36 ± 0.10	0.63	0.58 ± 0.35	0.58 ± 0.22	0.99	1.06 ± 0.30	0.39 ± 0.11	<0.01
Leucine	0.96 ± 0.04	0.87 ± 0.12	0.15	0.70 ± 0.13	0.75 ± 0.06	0.42	1.06 ± 0.30	1.65 ± 0.13	<0.01
Lysine	1.07 ± 0.08	0.93 ± 0.11	0.07	0.89 ± 0.18	1.01 ± 0.07	0.13	1.09 ± 0.27	1.37 ± 0.20	0.03
Proline	0.42 ± 0.04	0.40 ± 0.06	0.62	0.35 ± 0.08	0.38 ± 0.02	0.32	0.50 ± 0.08	0.77 ± 0.09	<0.01

[1]The statistical analysis between female and male groups. [2]The statistical analysis between green shelled eggs and white-shelled eggs groups.

Table S7. The threshold information of HT selective sweep analyses.

Statistics	HT vs. MDN	LW vs. MDN	JD vs. MDN	HT vs. YX
F_{ST}	0.773	0.81	0.791	0.625
Tajima's D	-1.498	-1.504	-2.149	-1.498
iHS	-2.410	-2.279	-2.402	-2.410
XP-EHH	3.368	3.961	2.983	2.789
